# DECADE-pilot: decision aid, action planning, and follow-up support for patients to reduce the 10-year risk of cardiovascular diseases—a protocol of a randomized controlled pilot trial

**DOI:** 10.1186/s40814-017-0172-5

**Published:** 2017-08-08

**Authors:** Iris Tinsel, Achim Siegel, Claudia Schmoor, Anika Buchholz, Wilhelm Niebling

**Affiliations:** 1 Division of General Practice, Medical Center—University of Freiburg, Faculty of Medicine, Elsässerstr. 2m, 79110 Freiburg, Germany; 2 Clinical Trials Unit, Faculty of Medicine, Medical Center—University of Freiburg, Elsässerstr. 2, 79110 Freiburg, Germany; 3 Institute of Medical Biometry and Epidemiology, University Medical Center Hamburg-Eppendorf, Martinistraße 52, 20246 Hamburg, Germany

**Keywords:** Cardiovascular diseases, Cardiovascular risk, Lifestyle changes, Behavior change, Decision aids, Patient activation, Self-management, Primary care, Family medicine

## Abstract

**Background:**

A healthy lifestyle can reduce cardiovascular risk (CVR) and prevent premature death. Usually most patients at increased CVR have difficulties implementing the necessary health behavior changes, such as smoking cessation, increasing of physical activity, healthy diet, stress reduction, etc. In this pilot study, a new intervention (DECADE) that includes a cardiovascular risk calculation, evidence-based decision aids, action planning, and follow-up support for patients to reduce their 10-year risk of cardiovascular diseases will be tested in primary care. The objectives of this trial are to test (1) the feasibility of the study design in preparation for the main trail including (2) the usability and acceptance of DECADE, and (3) initial data to ascertain that changes can be observed in these patients.

**Methods:**

This randomized controlled pilot trial will generate initial data on the potential effects of DECADE on patients’ self-evaluated activity and behavior change as well as on clinical outcomes such as blood pressure, cholesterol, body mass index (BMI), HbA1C, and CVR score. In the qualitative part of the study, we will analyze data collected in semi-structured interviews with participating general practitioners (GP) and in patient questionnaires.

**Discussion:**

The outcomes of this pilot study will indicate whether DECADE is a promising intervention in the domain of patient-centered prevention of cardiovascular diseases (CVD) and whether a larger multi-center randomized controlled trial is feasible.

**Trial registration:**

German Clinical Trials Register (DRKS), DRKS00010584

**Electronic supplementary material:**

The online version of this article (doi:10.1186/s40814-017-0172-5) contains supplementary material, which is available to authorized users.

## Background

Cardiovascular diseases (CVD) are the most frequent causes of death worldwide [1] as well as in Germany [2]. In addition to a genetic predisposition, an unhealthy lifestyle, such as smoking, sedentary life, unhealthy diet, critical or risky alcohol consumption, often together with adverse psychosocial factors (e.g., a high stress level or mental diseases such as depression), can lead to hypertension, obesity, diabetes, high cholesterol, and finally arteriosclerosis, which in turn increase the individual’s cardiovascular risk (CVR) [1, 3, 4]. Behavioral risk factors are responsible for about 80% of coronary heart and cerebrovascular diseases worldwide [1]. A healthy lifestyle can reduce CVR and prevent premature death [5]. Most patients with increased CVR do not implement the necessary health behavior changes or have difficulties maintaining a healthier lifestyle [6, 7]. This is particularly true for smoking cessation because of the high rate of nicotine dependence [8]. But also changes regarding regular physical activity or weight reduction often have low success rates [9]. As CVD are among the most frequent causes of treatment in primary care [10, 11], lifestyle-related advice is of crucial importance in primary and secondary prevention in general practice [12].

There are various interventions aiming at CVR reduction, among them a large number of decision aids (DAs) for patients, which are found in special databases or on websites [13–15]. Most of these DAs target decisions about taking specific medications, e.g., oral anticoagulants, statins, and oral antihyperglycaemic agents, while some help decide on hypertensive medication, lifestyle changes, or specific surgeries. DAs may be web based or paper based, and some of them include CVR calculators. In general, the listed DAs cover one or two risk factors and decision options but do not consider the complexity of risk factors and treatment options. An exception is the German CVR calculator Arriba [16]: with this computer-based tool, general practitioners (GPs) calculate the CVR of the patient and the possible absolute risk reduction of different treatment options (smoking cessation, physical activity, healthy diet, and the intake of antihypertensive drugs, statins, anticoagulation, or antidiabetic drugs). The calculations are the basis for shared decision-making (SDM). Other interventions to reduce the CVR involve patient or health professional education in SDM [17, 18]. Another patient-centered complex intervention in Switzerland included structured follow-up consultations [19]. Most interventions were evaluated positively regarding patient satisfaction and usability [19–22], and educational training enhanced knowledge [17], and was evaluated mainly positively by the GPs [19, 23]. In general, however, interventions targeting SDM did not have significant effects on patients’ health behavior or on clinical outcomes [17, 20, 21, 24–26]. Instead, programs to enhance patients’ self-management—especially in combination with a booster session or follow-up consultations—showed positive effects on patients’ behavior changes [19, 27–31]. However, according to a review of self-care programs by Spenceley et al. [31], patients with diabetes feel that they are faced with (too) high expectations from their GPs but at the same time feel that they do not receive enough support from their GPs. According to these authors, the identified barriers result from a disease centered instead of a patient-centered concept. Nevertheless, investigations on disease management programs (DMPs) for patients with diabetes and coronary heart disease showed a tendency towards positive effects on mortality, process parameters (e.g., behavior change), and surrogate parameters (e.g., cholesterol or HbA1c values) [32, 33]. A review and a meta-analysis showed that patients with diabetes participating in DMPs receive better diagnostics and examinations [32], as well as higher contact frequencies with the GP, which finally have a positive impact on DMP efforts [33].

The example of the diabetes DMP shows that structured care including follow-up visits has a positive impact on patient health. Patients’ barriers could be reduced by adopting more patient-centered care and a stepwise health behavior change. But DMPs exist neither for all cardiovascular diseases nor in primary prevention. At the same time, investigations showed that patient participation in health-related behavior decisions and/or support has generally not been sufficiently considered. General practitioners justify this with lack of time but also with patient factors like low adherence [34–36].

Staff members of the Division of General Practice within the Medical Center of the University of Freiburg have developed a new intervention called “DECADE.” DECADE provides an evidence-based decision aid, action planning, and follow-up support for patients to reduce the 10-year risk of cardiovascular diseases. The development was conducted using an iterative improvement process: eleven experts—GPs, psychologists, medical doctors specializing in nutrition, physical medicine or sports medicine and health scientists from other departments and institutions—as well as eight outpatients assessed DECADE in interviews, group discussions, and in written form. Eight experts and five patients took part in a standardized survey prior to the last revision of DECADE. Details of the development process of DECADE have been published in the German Clinical Trials Register (DRKS https://www.drks.de/drks_web/navigate.do?navigationId=trial.HTML&TRIAL_ID=DDRKS00003554) and in conference papers [37, 38]).

The overall concept of DECADE is predicated on the principles of evidence-based medicine, patient orientation, and self-management. We used the approach of shared decision-making (SDM) [39, 40], which often includes the use of decision aids [20]. To meet the requirements of long-term patient support, we integrated the concept of the health action process approach (HAPA). HAPA states that the health behavior changes must be understood as a process consisting of motivation and volition phases containing action plans and action control, which is influenced by risk perception, outcome expectancies, self-efficacy, situational barriers, and support [41, 42]. Furthermore, we considered the principle of small steps to facilitate behavioral changes [12, 19] as well as structured follow-up sessions [28].

DECADE contains:

1. Two printed booklets with (a) evidence-based information about CVDs, physical activity, diet, weight, blood pressure, hypertension, cholesterol, blood glucose, smoking, alcohol, stress, sleep deprivation, psychological comorbidities, and medical treatment, (b) decision aids, (c) self-monitoring elements, such as protocols for physical activity, diet, etc., action planning and goal attainment scales, as well as (d) a glossary. The DECADE booklets have a modular design. Therefore, DECADE can be applied to patients who have one or more different risk factors for CVD. DECADE—as a low-threshold intervention—should reach patients in primary and secondary CV prevention.
2. Patients using DECADE have password-protected access to a set of links on our homepage, which offer further information about CVR and CVD from established organizations, such as the German Agency for Quality in Medicine (äzq), the Institute for Quality and Efficiency in Healthcare (IQWiG), the National Association of Statutory Health Insurance Physicians (KBV), the Federal Center for Health Education (BZgA), and Independent Patient Consulting Germany (UPD). Listed topics are evidence based and detailed patient information regarding general health, smoking and alcohol, CVD, metabolic disorders, self-help groups, etc.
3. The implementation of DECADE in primary care includes structured consultations by the GPs. They start with (a) the calculation of the patient’s CVR using the Arriba calculator [16, 22], followed by (b) shared decision making regarding treatment and goal setting, (c) support for individual action planning and self-monitoring, and finally, (d) a second CVR calculation after 4 months.

Based on a search for comparable interventions in national and international decision aid databases [13, 14, 43], we could not find other interventions that include all of the elements offered by DECADE.

The aims of this pilot study are first to test DECADE regarding its usability and acceptance in primary care. Second, we will test the feasibility of the randomized study design. Third, the pilot study will generate initial data on the potential effects of DECADE on “patient activation” in terms of patient knowledge, skills, confidence, and behavior critical for coping with a chronic illness [44], behavioral changes, and clinical outcomes. The evaluation of DECADE will provide information about any necessary revisions of the intervention and the study design of a planned multi-center trial, and finally on the possibility of implementing DECADE as part of a patient-centered prevention of CVD.

## Methods/design

### Design and setting of the study

The study will be conducted as a randomized controlled pilot trial with six GP practices in the South Baden region of Germany. Patients with at least one cardiovascular risk factor (hypertension, hypercholesterolemia, diabetes, arteriosclerosis, smoking, obesity, high stress level or drug prescription against hypertension, high cholesterol, or diabetes) will be invited to take part in the study. Each GP practice will seek to include 15 patients in the study. After a baseline data assessment (T0), patients will be randomized into two groups: the Arriba group and the DECADE group. GPs calculate the individual CVR and possible treatment effects of each patient in both groups by using the Arriba calculator [16]. Patients of the DECADE group additionally receive the DECADE booklets. A follow-up assessment (T1) is scheduled 4 months after the baseline assessment. Patients of the Arriba group will receive the DECADE booklets after finishing the study (waiting list control design).

### Study population and recruitment procedure

For this pilot study, *GPs* associated with the Department of General Practice of the University Medical Center Freiburg (Germany) were informed about the DECADE pilot study in the context of network meetings and training courses. Additionally, GPs who cooperated with the Department of General Practice of the University Medical Center Freiburg in earlier studies received written information about the study. Interested GPs who attend to more than 500 patients in a quarter of a year and guarantee the full range of medical care were introduced to the DECADE pilot study. The introduction included a practical application of the CVR calculator Arriba if necessary and a detailed explanation of the aims and use of the DECADE booklets, as well as of the structured consultations and performance of the study. GPs who ultimately wanted to participate signed an informed consent form. More than six GP practices were interested in taking part in the study. Therefore, a waiting list was compiled. In case of GP drop-outs, GP practices from the waiting list will be included.

Inclusion criteria: Female and male adult patients of the participating GP practices with at least one risk factor for CVD, such as hypertension, hypercholesterolemia, diabetes, arteriosclerosis, smoking, obesity, and high stress level; patients with drug prescription for high blood pressure, high cholesterol, or diabetes.

Exclusion criteria: (1) Pregnancy, (2) substance or alcohol dependence, severe eating disorder, (3) considerable cognitive impairment or mental disease, (4) seriously debilitating disease or short life expectancy, (5) actual or planned participation in a rehabilitative measure, or (6) insufficient German language skills (spoken or written).

To assure a random sample of the patients in each GP practice, medical assistants (MAs) of the practices are asked to apply a structural recruitment procedure: MAs check the electronic medical records (EMR) of adult patients who are scheduled during two morning hours of 2 days and two afternoon hours of a third day in a week regarding CVR factors. If at least one of the abovementioned CVR factors is found in the EMR and if those patients fulfill the inclusion criteria, they will be informed about the study. If they are willing to take part in the study, they sign the study’s informed consent form. The GP practices are instructed not to recruit patients who live in the same household to prevent a contamination of the control group.

**Fig. 1:**
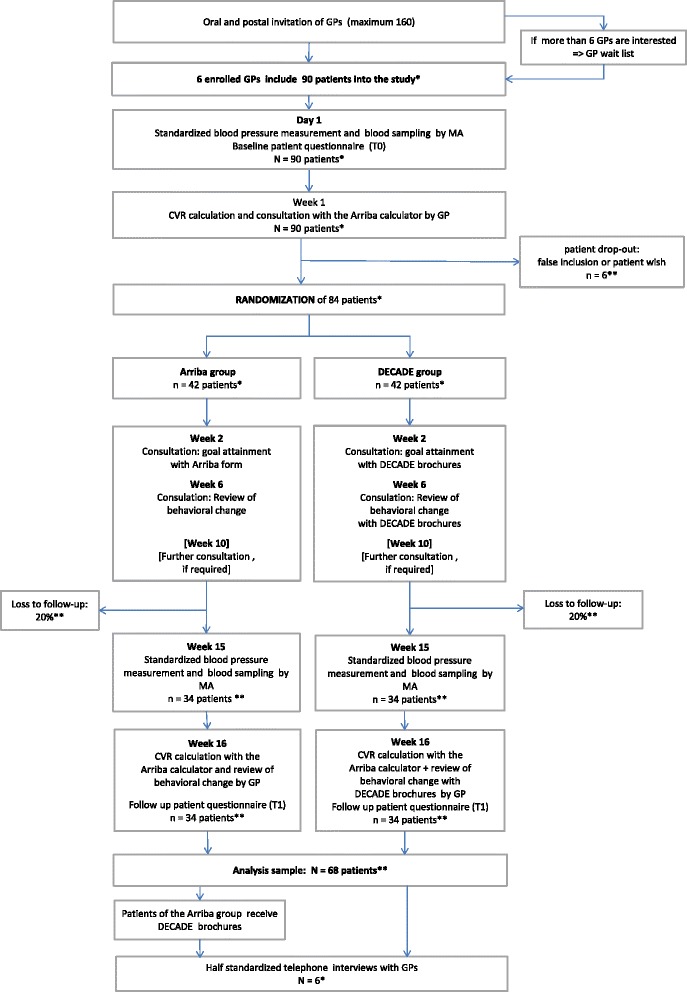
Flowchart of the DECADE pilot study. *GP* general practitioner, *MA* medical assistant, *CVR* cardiovascular risk. *One asterisk* indicates target numbers. *Double asterisks* indicate estimated numbers

Included patients fill out the baseline questionnaire (T0). In order to calculate the CVR with Arriba, MAs take a blood sample to determine necessary laboratory values (total and HDL cholesterol, and in the case of diabetes HbA1c) and perform a standardized blood pressure measurement (mean of three measurements after a 5-min resting period) of all patients. A few days later, the GPs use the Arriba calculator to calculate the CVR and consult the patients regarding reasonable treatment options and the effect on the absolute individual CVR. All patients receive the printed Arriba form that contains clinical data, treatment options, and actual and potential CVR scores. The recruitment procedure and details of the T0 data assessment are presented in Fig. 1.

*Randomization of the patients* will be carried out by GPs after CVR calculation using prepared, sealed randomization envelopes. Randomization envelopes are prepared by the Clinical Trials Unit of the University Medical Center Freiburg for concealment of randomization. Randomization was stratified by GP practice, with half of the patients assigned to the DECADE group and half of the patients to the Arriba group. GPs do not know the result of the randomization before they open the randomization envelopes to avoid selection bias. Blinding during the intervention is not possible because (1) the GPs use the DECADE booklets together with their patients, (2) MAs distribute the patient questionnaires which differ in T1 according to the study group, and (3) patients are informed about the waiting list control design.

### Intervention

After randomization, patients allocated to the DECADE group will receive the DECADE booklets in addition to the printed Arriba form. The booklets contain evidence-based information about CVD and risk factors, mention possible actions to take to reduce these risk factors, and explain decision aids for each risk factor with the aim of supporting patients in making realistic decisions. Further, the booklets include materials which support the patient in defining goals with the GP and planning activities step by step. They additionally provide motivating impulses, weekly protocols, and goal attainment scales. Furthermore, patients of the DECADE group receive password-protected access to the DECADE information website [45]. The website provides links to established organizations that offer evidence-based and detailed patient information regarding general health, smoking and alcohol, CVD, metabolic disorders, self-help groups, and actions to take in case of emergency.

The follow-up consultations are carried out in the same chronical order in the DECADE group and the Arriba group: In the first week after the CVR has been calculated, patients reflect on possible treatment options. Together with their GP, they set their goals after 1 week. After a further 4 weeks, patients of both groups review their experiences regarding the implementation of agreed goals together with the GP. GPs advise the patients according to their needs. GPs are free to consult patients whenever patients have a high need for advice, e.g., in the 10th week. In the fourth month (T1), MAs take the second blood sample and a standardized blood pressure measurement. GPs calculate the CVR using the Arriba calculator and consult the patients. Afterwards, patients complete the second questionnaire (T1), and the study ends (see Fig. 1.)

Patients of the Arriba group receive the DECADE booklets and the corresponding structured consultations—if they are interested—after finishing the T1 questionnaire.

### Data collection

#### Quantitative data collection

##### Clinical data

The MAs submit pseudonymized copies of the Arriba forms to the project team of the DECADE pilot study (Division of General Practice, Medical Center-University of Freiburg). The forms contain gender, age, dichotomous data (yes/no) like smoking status, arteriosclerosis, genetic disposition for CVD, intake of antihypertensive drugs, and diabetes, systolic blood pressure (SBP) values, total and HDL cholesterol values, and in case of diabetes HbA1c values. Dichotomous data (yes/no) on reasonable treatment options include smoking cessation, physical activity, healthy diet, intake of statins, antihypertensive drugs, blood thinners and in case of diabetes metformin, and the possible overall effect on the individual CVR score.

#### Patient questionnaires

The baseline assessment (T0) includes the following data:

- Subjective quality of life (Visual Analogue Scale of the EQ-5D (EQ-VAS)) [46]
- Height and weight
- Self-rated risk factors like smoking, overweight, unhealthy diet, lack of physical activity, stress, and alcohol (Indicators of the Rehabilitation Status (IRES)) [47]
- Patient activation measure (German 13-item version; PAM-13D). It assesses patient knowledge, skills, confidence, and behavior critical for coping with a chronic illness [44].
- Sociodemographic data: gender, age, family status, educational level, occupational status, and extent of gainful employment

In the follow-up assessment (T1), the same data are collected as in the baseline assessment (except for sociodemographic data). In addition, the following are assessed:

- Changes in health behavior [48],
- Adapted Goal Attainment Scaling (adapted GAS) [49, 50] and satisfaction with goal attainment (our own development). On the adapted GAS, patients mark their individual treatment goals (such as various lifestyle changes, regular blood pressure or blood glucose measurements at home, or regular intake of medication as prescribed) and rate whether they attained their goals on a scale from 0 (not attained at all) to 5 (better attained than I planned). The structure of the second scale (satisfaction with goal attainment) corresponds to the adapted GAS, but patients rate their satisfaction with goal attainment from 1 (very satisfied) to 4 (very unsatisfied).
- The usefulness scale for patient information (USE) [51],
- Satisfaction with consultation and support by the GP (our own development). This instrument includes six items, specifically on (1) the GP’s consultation on the individual CVR, (2) agreement on treatment goals, (3) communication about treatment plans or behavior changes, (4) communication about success and failure, (5) shared search for solutions in case of problems, and (6) motivation by the GP. Patients rate their satisfaction on a scale from 1 (very satisfied) to 4 (very unsatisfied).

#### Qualitative data collection

1. The follow-up questionnaire (T1) of the DECADE group contains open questions that are to evaluate the DECADE intervention in detail regarding (1) the arrangement and design of the booklets, (2) the intelligibility and amount of information, (3) patients’ usage of the materials, (4) satisfaction with the DECADE booklets, and (5) suggestions for improvement.
2. After data assessment in T1 is finished (last patient out), participating GPs will be asked in a semi-structured telephone interview about their experiences during the implementation and about patient acceptance of DECADE. The topics of the interview are available in the supplemented material (Additional file 1).

#### Data management

All data will be submitted and recorded in the Division of General Practice, Medical Center—University of Freiburg, Faculty of Medicine.

Pseudonymized quantitative patient data will be merged into an SPSS data file (IBM SPSS; Version 23).

Data management will check patient data regarding legal age and existing CVR factors. Furthermore, the data management will ask the GP practices whether some included patients live in the same household. If so, and if the corresponding patients belong to different treatment groups, the data management will exclude patients of the Arriba group from the statistical analyses.

Open answers written down in the patient questionnaires will be transferred into separate Word documents together with the patient’s pseudonym. The semi-structured GP interviews will be digitally recorded and transcribed using pseudonyms.

### Study endpoints and analyses

Our aim is to investigate the usability and acceptance of DECADE and determine whether improvements of the intervention, mode of implementation, study design, and endpoints are necessary for future evaluations.

To test our first study objective, the usability and acceptance of DECADE, we will analyze the qualitative data. This analysis will be performed by using the software MAXQDA (Version 11)[52]. All pseudonymized text material will be coded considering deductive categories (key questions of the patient questionnaire and the guideline-based GP interviews), and inductive categories (new aspects, if mentioned within the questionnaires or interviews). The text materials will be structured and validated by coding the interviewed person (patient or GP) and assigning positive, negative, or ambivalent validations of the categories in consideration of their context. The text analyses continue by counting the frequencies of evaluated codings [53, 54].

The second study aim, to test the feasibility of the study, will be reached by evaluating the study progress. This involves assessing the inclusion process, the randomization procedure, and the loss to follow-up of patients, as well as evaluating chosen instruments of the questionnaire, primary and secondary endpoints, and the analysis strategy.

The primary endpoint with regard to the third study aim is “patient activation,” as measured by a change in the PAM-13D score [44] between T0 and T1. The change in the PAM-13D score in the randomized groups and the potential effect of the intervention on this change will be estimated in a linear regression model with 95% confidence intervals, including as independent factors the intervention, GP practice, CVR score, and the PAM-13D score at T0. This will be done to facilitate sample size planning of the main trial.

Secondary endpoints with regard to the third study aim are the following: (1) The actual state of health, measured by a change in the EQ-VAS score [46] between T0 and T1. The potential effect will be estimated by linear regression including as independent factors the intervention, GP practice, CVR score, and EQ-VAS score at T0. The outcomes (2) change of health behavior [48], (3) the usefulness of patient material (USE) [51], (4) the adapted Goal Attainment Scaling (GAS) [49, 50], and (5) the satisfaction with goal attainment (own development) will be analyzed descriptively by the comparison of mean scores in T1.

Due to the randomization at the patient level, we can assume that sociodemographic and clinical data will be evenly distributed between the DECADE group and the Arriba group. Nevertheless, we will describe potential differences between both groups regarding age, gender, educational level, and occupational status, as well as health status (clinical data and patient reported outcomes) at baseline. The relationship of baseline characteristics with the primary endpoint patient activation (PAM-13D) will be described. Potential factors identified as prognostic will be considered in the planned multi-center trial. Additionally, the change in the clinical data of SBP, total and HDL cholesterol, body mass index (BMI), HbA1C, and CVR score will be analyzed descriptively by the change of mean values between T0 and T1.

Before carrying out the analyses, possible missed patterns will be explored (frequencies as well as relationship to patient characteristics and outcome measurements). If necessary, single items have to be excluded from the analyses. Imputation is not planned in this pilot study.

### Sample size calculation

As the focus of this pilot study is on usability and the acceptance of the DECADE intervention, no formal sample size calculation was performed. Sample size considerations are based on feasibility of recruitment within a reasonable timeframe. Nevertheless, we think that we can observe trends in the quantitative analyses.

We expect that the number of patients who drop out immediately after the randomization will be low. One reason for this assumption is the waiting list control design of the study. We assume that each of the six GPs will include 15 patients (total *N* = 90) and that 84 patients will take part in the study (42 in the Arriba group and 42 in the DECADE group). We further estimate a loss to follow-up between T0 and T1 of 20%. This conservative estimate is based on drop-out rates between 10 and 60% in trials similar to our pilot study regarding the target group [18, 55], the CVR consultation with Arriba [22], and the intervention [19, 21]. Thus, we expect that the analysis sample will contain *N* = 68 patients. With 34 patients in each study arm, the primary endpoint (PAM-13D) can be estimated—assuming a standard deviation of 15 [44]—with a precision of about 5 (half the length of the 95% confidence interval).

## Discussion

Main objectives of this pilot study are (1) to test the new intervention DECADE in terms of its usability and acceptance in primary care, (2) to test the feasibility of the study design and analysis strategy, and (3) to generate initial data on the potential effects of the DECADE intervention compared to the Arriba intervention.

We will take into account potential selection bias regarding patient age, gender, and CVR factors. Therefore, we will compare these characteristics of invited but non-participating patients versus included patients. The anonymized data of non-participants will be assessed by the MAs and submitted to the project team of DECADE pilot.

The comprehensive evaluation of the DECADE pilot study will combine the results of the quantitative and qualitative analyses and will indicate whether DECADE is a promising intervention in the domain of patient-centered prevention of CVD. We will use the results to refine the DECADE intervention and the planned multi-center randomized controlled trial.

### Trial status

The enrollment for the DECADE pilot study started in July 2016. Recruitment is still in progress. Quantitative and qualitative data assessment will be finished approximately in February 2017.

## Additional files

Additional file 1: Semi-structured telephone interviews with general practitioners. List of topics. (PDF 75 kb)

## Abbreviations

BMI: Body mass index
CV: Cardiovascular
CVD: Cardiovascular diseases
CVR: Cardiovascular risk
DMP: Disease management program
DRKS: German register of clinical studies
EMR: Electronic medical records
EQ-VAS: EuroQol Visual Analogue Scale
GAS: Goal attainment scaling
GP: General practitioner
HAPA: Health action process approach
HbA1c: Hemoglobin A1c
HDL cholesterol: High-density lipoprotein cholesterol
IRES: Indicators of the Rehabilitation Status
MA: Medical assistant
PAM: Patient activation measure
SDM: Shared decision-making
SBP: Systolic blood pressure
USE: Usefulness scale for patient information
