# Supplementary material for: DECADE-pilot: decision aid, action planning, and follow-up support for patients to reduce the 10-year risk of cardiovascular diseases—a protocol of a randomized controlled pilot trial
Source: Pilot Feasibility Stud. 2017 Aug 8;3:32. doi: 10.1186/s40814-017-0172-5 (PMC5549435; doi:10.1186/s40814-017-0172-5)
Supplement: Supplementary file 1 — Semi-structured telephone interviews with general practitioners. List of topics. (PDF 75 kb) [file 40814_2017_172_MOESM1_ESM.pdf]

Semi-structured telephone interviews with participating general practitioners

List of interview topics:

- GPs experiences with patient recruitment.
- GPs experiences with the structured consultations: Arriba consultation, implementation of the DECADE brochures, shared decision making and goal setting, support for action planning and self-monitoring.
- Patients' feedback about the structured consultations and in the DECADE group about the DECADE brochures (modular structure, design, information, usability, motivation), and the DECADE website.
- GPs perceived differences between the DECADE group and the Arriba group as well as differences on patient level. This refers to goal setting and action planning, health behavior changes, motivation or frustration.
- Mean time needed for the different consultations.
- General satisfaction with the structured consultations, the DECADE brochures and the global concept of DECADE.
- Improvement suggestions.
